# Standard-dose versus double-dose dolutegravir in HIV-associated tuberculosis in South Africa (RADIANT-TB): a phase 2, non-comparative, randomised controlled trial

**DOI:** 10.1016/S2352-3018(23)00081-4

**Published:** 2023-05-22

**Authors:** Rulan Griesel, Ying Zhao, Bryony Simmons, Zaayid Omar, Lubbe Wiesner, Claire M Keene, Andrew M Hill, Graeme Meintjes, Gary Maartens

**Affiliations:** a Division of Clinical Pharmacology, Department of Medicine, University of Cape Town, Cape Town, South Africa; b Wellcome Centre for Infectious Diseases Research in Africa, Institute of Infectious Disease and Molecular Medicine, and Department of Medicine, University of Cape Town, Cape Town, South Africa; c Department of Medicine, University of Cape Town, Cape Town, South Africa; d LSE Health, London School of Economics and Political Science, London, UK; e Médecins Sans Frontières, Cape Town, South Africa; f Centre for Tropical Medicine and Global Health, Nuffield Department of Medicine, University of Oxford, Oxford, UK; g Department of Pharmacology and Therapeutics, University of Liverpool, Liverpool, UK

## Abstract

**Background:**

The drug–drug interaction between rifampicin and dolutegravir can be overcome by supplemental dolutegravir dosing, which is difficult to implement in high-burden settings. We aimed to test whether virological outcomes with standard-dose dolutegravir-based antiretroviral therapy (ART) are acceptable in people with HIV on rifampicin-based antituberculosis therapy.

**Methods:**

RADIANT-TB was a phase 2b, randomised, double-blind, non-comparative, placebo-controlled trial at a single site in Khayelitsha, Cape Town, South Africa. Participants were older than 18 years of age, with plasma HIV-1 RNA greater than 1000 copies per mL, CD4 count greater than 100 cells per μL, ART-naive or first-line ART interrupted, and on rifampicin-based antituberculosis therapy for less than 3 months. By use of permuted block (block size of 6) randomisation, participants were assigned (1:1) to receive either tenofovir disoproxil fumarate, lamivudine, and dolutegravir plus supplemental 50 mg dolutegravir 12 h later or tenofovir disoproxil fumarate, lamivudine, and dolutegravir plus matched placebo 12 h later. Participants received standard antituberculosis therapy (rifampicin, isoniazid, pyrazinamide, and ethambutol for the first 2 months followed by isoniazid and rifampicin for 4 months). The primary outcome was the proportion of participants with virological suppression (HIV-1 RNA <50 copies per mL) at week 24 analysed in the modified intention-to-treat population. This study is registered with ClinicalTrials.gov, NCT03851588.

**Findings:**

Between Nov 28, 2019, and July 23, 2021, 108 participants (38 female, median age 35 years [IQR 31–40]) were randomly assigned to supplemental dolutegravir (n=53) or placebo (n=55). Median baseline CD4 count was 188 cells per μL (IQR 145–316) and median HIV-1 RNA was 5·2 log_10_ copies per mL (4·6–5·7). At week 24, 43 (83%, 95% CI 70–92) of 52 participants in the supplemental dolutegravir arm and 44 (83%, 95% CI 70–92) of 53 participants in the placebo arm had virological suppression. No treatment-emergent dolutegravir resistance mutations were detected up to week 48 in the 19 participants with study-defined virological failure. Grade 3 and 4 adverse events were similarly distributed between the study arms. The most frequent grade 3 and 4 adverse events were weight loss (4/108 [4%]), insomnia (3/108 [3%]), and pneumonia (3/108 [3%]).

**Interpretation:**

Our findings suggest that twice-daily dolutegravir might be unnecessary in people with HIV-associated tuberculosis.

**Funding:**

Wellcome Trust.

## Introduction

WHO's preferred first-line antiretroviral therapy (ART) regimen for adults and adolescents with HIV is the second-generation integrase strand transfer inhibitor dolutegravir, combined with tenofovir and lamivudine or emtricitabine.^1^ A disadvantage of dolutegravir is substantial drug–drug interaction with rifampicin,^2^ which is important as tuberculosis is the most common cause of hospitalisation and mortality among people living with HIV.^3^, ^4^ A previous study of healthy volunteers showed that increasing the dose of dolutegravir from 50 mg daily to 50 mg every 12 h can overcome the effect of rifampicin induction of genes important in the metabolism and transport of dolutegravir.^5^ Twice-daily dolutegravir was effective and well tolerated in a non-comparative, active control, randomised trial in people with HIV with tuberculosis.^6^

Implementing an additional dose of dolutegravir with antituberculosis therapy is challenging in high-burden settings, illustrated by the finding of a Botswanan study in which standard-dose dolutegravir was used in almost half of patients on dolutegravir-based ART during antituberculosis therapy, despite national guidelines recommending double-dose dolutegravir.^7^ In high-burden settings, HIV and tuberculosis are often managed in separate clinics and clinic staff might not be aware that patients are being treated for both diseases. Furthermore, dolutegravir-based combination ART is dispensed as fixed-dose combination tablets, which increases the risk of dolutegravir single-tablet stockouts. There is some evidence that standard-dose dolutegravir with rifampicin-based antituberculosis therapy might be effective. A phase 2b dose-ranging study among treatment-naive people living with HIV reported similar high rates of virological suppression with all the tested doses of dolutegravir (10 mg, 25 mg, and 50 mg).^8^ Additionally, dolutegravir shows avidity to the integrase receptor with a dissociative half-life of 71 h,^9^ which might mitigate against the emergence of resistance with transient low dolutegravir trough concentrations. Furthermore, the median dolutegravir trough concentration when dosed at 50 mg daily with rifampicin was reduced by 85% but was still 2·4 times above the protein-adjusted inhibitory concentration (PA-IC_90_) in a study of healthy volunteers.^2^ Finally, standard-dose dolutegravir treatment resulted in similar rates of virological suppression as twice-daily dolutegravir treatment in a large retrospective cohort study.^7^

**Research in context**

**Evidence before this study**

We searched PubMed for clinical studies on the dosing of dolutegravir with rifampicin-based antituberculosis therapy published between Jan 1, 2010, and Oct 10, 2022, using the search terms (“tuberculosis” AND “dolutegravir” AND “HIV”) with no language restrictions. Our search yielded one non-comparative randomised trial and one retrospective observational cohort study. INSPIRING was a non-comparative, active control, randomised, open-label study among people living with HIV who were naive to antiretroviral therapy (ART) with CD4 counts of at least 50 cells per μL. Participants were randomly assigned to receive either dolutegravir-based or efavirenz-based ART. Dolutegravir was dosed at 50 mg twice daily during and for 2 weeks after antituberculosis therapy. Week 48 virological suppression (HIV-1 RNA <50 copies per mL) in INSPIRING was reported in 75% (52/69) of participants in the supplemental dolutegravir group and 82% (36/44) of participants in the efavirenz group. These results support the hypothesis that twice-daily dolutegravir is effective for people with HIV who are on rifampicin-based antituberculosis therapy. Twice-daily dolutegravir was well tolerated. Although INSPIRING generated high-quality evidence, the study was not powered for between-arm comparisons. A retrospective observational cohort study in Botswana reported on the viral load suppression rates achieved with dolutegravir-based ART in people with HIV who were co-infected with tuberculosis under programmatic conditions. A high proportion of patients (322 [44%] of 739) received once-daily dolutegravir instead of the twice-daily dosing recommended in their national guidelines, which indicates the difficulty of implementing supplemental dolutegravir dosing in a high-burden setting. Virological suppression, which was determined while participants were on antituberculosis therapy, was reported in 204 (95%) of 214 individuals in the once-daily dosing group and in 241 (95%) of 254 individuals in the twice-daily dosing group. The risk of bias is high in retrospective cohort studies but the relatively large sample size and the almost identical viral suppression rates in the two dolutegravir dosing groups suggest that supplemental dolutegravir dosing might be unnecessary for people with HIV on rifampicin-based antituberculosis therapy.

**Added value of this study**

To our knowledge, RADIANT-TB is the first randomised trial to assess the need for twice-daily dosing with dolutegravir when co-administered with rifampicin-based antituberculosis therapy. We found similar rates of virological suppression in both dolutegravir dosing arms. None of the participants who had study-defined virological failure developed treatment-emergent integrase strand transfer inhibitor resistance and no major safety issues were reported in either arm. Our findings suggest that twice-daily dolutegravir dosing with rifampicin-based antituberculosis therapy might be unnecessary, but we did not have the power to compare outcomes by study arm.

**Implications of all the available evidence**

The findings of our study together with those of the Botswanan retrospective cohort study suggest that once-daily dolutegravir is as effective as twice-daily dolutegravir for virological suppression in patients on rifampicin co-treatment for tuberculosis.

We aimed to determine whether virological outcomes with standard-dose dolutegravir-based ART would be acceptable in people with HIV on rifampicin-based antituberculosis therapy.

## Methods

### Study design and participants

RADIANT-TB was a phase 2b, randomised, double-blind, non-comparative, placebo-controlled trial at a single clinical site in Khayelitsha, Cape Town, South Africa. National regulatory and ethical approval (Human Research Ethics Committee of the University of Cape Town) were obtained before commencing the study. The detailed study protocol plan has been published online.^10^

Patients were referred to the study from three primary care clinics in Khayelitsha. Inclusion criteria were age older than 18 years, seropositivity for HIV-1, plasma HIV-1 RNA greater than 1000 copies per mL, CD4 count greater than 200 cells per μL (subsequently changed to more than 100 cells per μL), ART naivety or first-line ART interruption (with ART duration <6 months or HIV-1 RNA <50 copies per mL <6 months before interruption), and being on rifampicin-based antituberculosis therapy for less than 3 months. Women of childbearing potential were placed on appropriate contraceptives for the duration of the study. Among the exclusion criteria were alanine aminotransferase more than three times the upper limit of normal, allergy or intolerance to one of the drugs in the regimen, and an active psychiatric condition or substance abuse judged likely to affect adherence. All participants gave written informed consent (in English or isiXhosa) before enrolment. Sex was self-reported, selecting either a male or female option.

### Randomisation and masking

By use of permuted block (block size of 6) randomisation, participants were assigned (1:1) to either receive oral tenofovir disoproxil fumarate 300 mg daily, oral lamivudine 300 mg daily, and oral dolutegravir 50 mg daily plus supplemental 50 mg dolutegravir 12 h later or tenofovir disoproxil fumarate, lamivudine, and dolutegravir plus matched placebo 12 h later. Randomisation was stratified by baseline ART status (ART-naive *vs* first-line ART interruption).

Study pharmacists used sequentially drawn individually sealed opaque envelopes to assign a treatment arm when dispensing medication. Allocation concealment was maintained as only the study pharmacists retained access to the randomisation schedules through a locked storage unit and strict access control. Participants and investigators remained masked for the duration of the study. Masking was achieved using tablets with an identical appearance. The study statistician had access to the randomisation schedules for 6-monthly data and safety monitoring committee meetings.

### Procedures

Participants received standard antituberculosis therapy (rifampicin, isoniazid, pyrazinamide, and ethambutol [oral daily weight-based method] for the first 2 months followed by rifampicin and isoniazid [oral daily weight-based method] for the subsequent 4 months) provided by the local tuberculosis programme. The duration of maintenance therapy was extended if necessary (skeletal or CNS tuberculosis, poor resolution of tuberculosis symptoms, poor adherence, or delayed sputum smear conversion) by local tuberculosis clinics. ART was initiated 8–12 weeks after initiation of antituberculosis therapy, except for a 5-month period during the early COVID-19 pandemic, when national treatment guidelines recommended ART initiation within 2 weeks of starting antituberculosis therapy, regardless of CD4 count. Participants were instructed to take the single oral tenofovir disoproxil fumarate 300 mg daily, oral lamivudine 300 mg daily, and oral dolutegravir 50 mg daily tablets with food in the morning and the 50 mg dolutegravir or matching placebo orally with food 12 h later. The supplemental dolutegravir or placebo was continued for 2 weeks after antituberculosis therapy was stopped. Dolutegravir 50 mg (Myltega) and matching placebo were donated by Mylan Pharmaceuticals (Canonsburg, PA, USA). Participants were asked to return all unused study medication for collection and counting by study staff. Co-trimoxazole preventive therapy was supplied by the local tuberculosis programme (trimethoprim [160 mg] and sulfamethoxazole [800 mg] daily, taken orally).

Tuberculosis diagnoses were made by local tuberculosis clinics and classified as microbiological or histological (acid-fast bacilli on sputum, Xpert MTB/RIF [Cepheid, Sunnyvale, CA, USA] or positive culture from any site, or histological diagnosis) or clinical or radiological (or both). Tuberculosis was classified as either pulmonary, extrapulmonary, or disseminated.

Screening procedures were HIV-1 RNA testing, CD4 cell count (if no result <3 months old available), safety laboratory testing, and clinical assessment. Plasma HIV-1 RNA viral load was determined with the Abbott Alinity M HIV-1 test (Abbott, Abbott Park, Green Oaks, IL, USA; lower limit of detection 20 copies per mL). If the screening CD4 count was between 50 cells per μL and 99 cells per μL the test was repeated when ART was planned to start to reassess eligibility. Woman of childbearing potential had a urinary pregnancy test.

Participants were enrolled within 8 weeks of the screening visit. Follow-up visits occurred at weeks 4, 8, 12, 16, 20, 24, and 48. Safety laboratory tests and plasma HIV-1 RNA were done at weeks 4, 8, 12, 24, and 48. CD4 counts were repeated at weeks 24 and 48. Plasma for dolutegravir trough concentrations and dried blood spots for tenofovir diphosphate were taken at weeks 4, 8, 12, 24, and 48. Pharmacokinetic samples were analysed by the Clinical Pharmacology Laboratory, University of Cape Town (Cape Town, South Africa) with accredited methods.^11^, ^12^

Participants with unsuppressed HIV-1 RNA (≥50 copies per mL) received adherence counselling and repeat HIV-1 RNA sampling every 4 weeks until suppression or study-defined virological failure (defined as HIV-1 RNA >1000 copies per mL at week 24 or if HIV-1 RNA was <50 copies per mL and then rebounded to >1000 copies per mL at any timepoint). Genotypic antiretroviral resistance testing was done on participants with study-defined virological failure (Applied Biosystems HIV-1 Genotyping kit; ThermoFisher Scientific, Applied Biosystems; Waltham, MA, USA). Resistance was defined as potential low-level, intermediate-level, or high-level resistance according to the Stanford HIV Drug Resistance Database. We also did genotypic antiretroviral resistance testing on stored baseline samples if integrase strand transfer inhibitor resistance was detected.

### Outcomes

The primary outcome was proportion of participants with virological suppression, defined as HIV-1 RNA less than 50 copies per mL at week 24, analysed in the modified intention-to-treat population and according to the US Food and Drug Association snapshot approach, which defines failure as any one of the following: HIV-1 RNA of 50 copies per mL or greater, missing HIV-1 RNA within the window period (±16 days), or if ART is discontinued. We did not regard switching of tenofovir or lamivudine for intolerance as a failure.

Secondary outcomes included proportion of participants with HIV-1 RNA of less than 50 copies per mL at weeks 12 and 48, analysed in the modified intention to treat and per-protocol populations; change in CD4 count from baseline to weeks 24 and 48; proportion of participants with dolutegravir trough concentrations above the PA-IC_90_ at weeks 4, 24, and 48; ART adherence assessment determined by tenofovir diphosphate in dried blood spots at weeks 12, 24, and 48; occurrence of grade 3 and 4 and serious adverse events; change in modified mini screen (MMS)^13^ and Insomnia Severity Index (ISI)^14^ questionnaires from baseline; adverse events requiring discontinuation of any drug in the ART regimen; the emergence of integrase strand transfer inhibitor resistance mutations in participants with study-defined virological failure; and all virological endpoints stratified by baseline ART-naive or first-line ART interruption status.

Adverse events were recorded at every visit and graded according to the Division of AIDS (DAIDS) adverse event grading tables.^15^ Only grade 3 and 4 (clinical and laboratory) and serious adverse events were captured. Participants were actively screened for the development of immune reconstitution inflammatory syndrome events by use of standard definitions.^16^ Screening for neuropsychiatric adverse events was done with the MMS^13^ and ISI^14^ questionnaires. The MMS scoring questionnaire covers symptoms for major depression, dysthymia, suicidality, hypomania, panic, agoraphobia, social phobia, obsessive compulsive disorder, post-traumatic stress disorder (PTSD), psychosis, and generalised anxiety. We removed two PTSD-related questions from the MMS for analysis purposes, as these events could have been present before enrolment. The MMS questionnaire was done at baseline and at weeks 12, 24, and 48. The ISI is a cumulative score of 28 insomnia-related questions. A score of 0−7 is classified as no clinically significant insomnia, 8−14 as subthreshold insomnia, 15−21 as moderate clinical insomnia, and 22−28 as severe clinical insomnia. The ISI questionnaire was done at baseline, at 4-weekly intervals until week 24, and at week 48.

### Statistical analysis

We assumed that 85% of participants would achieve virological suppression at week 24.^17^ With 49 participants per arm, the lower 95% CI of virological suppression at week 24 would exceed 70%, with a power of 80% and an a of 5% (one-sided test). We selected this lower 95% CI bound of virological suppression based on the outcomes from two randomised controlled trials with efavirenz-based ART in participants with HIV-associated tuberculosis, which reported virological suppression of 70% and 74% at 48 weeks.^18^, ^19^ Assuming a 10% rate of loss to follow-up, we aimed to enrol 54 participants per arm. The study was not powered for a formal between-arm efficacy comparison.

The primary outcome analysis was by modified intention to treat, which included all participants who received at least one dose of dolutegravir, and excluded those who switched from dolutegravir because of wishing to become pregnant, becoming pregnant, transfer out of the study for non-clinical reasons, and death from non-HIV-related and non-drug-related causes (as assessed by the study investigator). The per-protocol analysis included the same exclusions as modified intention to treat, but also excluded any participants lost to follow-up, missing an HIV-1 RNA measurement in the study visit window (±16 days around a visit date), and participants switched from dolutegravir for reasons other than failure of the regimen. The proportions of participants with virological suppression were determined with 95% CI. Between-group differences for secondary endpoints, where relevant, were analysed by use of χ^2^ test (or Fisher's exact test if the number in any cell was ≤5 or McNemar's test for paired observations) for categorical data and Wilcoxon rank sum test for continuous data.

Dried blood spot tenofovir diphosphate concentrations were used to measure adherence based on predefined concentration criteria for men and women.^20^ Samples with values below the lower limit of quantification (16 × 6 fmol/punch) were imputed as 50% lower limit of quantitation (8 × 3 fmol/punch). We did logistic regression adjusted for sex to assess the association of tenofovir diphosphate concentrations with virological suppression.

The time windows for dolutegravir trough concentrations were 12 h (±2 h) for participants on supplemental dosing with placebo or dolutegravir (defined as 12-h post-dose concentrations for the supplemental arm and 24-h post-dose concentrations for the placebo arm), and 24 h (±4 h; defined as 24-h post-dose concentrations in both arms). Participants still on supplemental dolutegravir or placebo at weeks 24 and 48 were excluded from analyses at these timepoints. Dolutegravir trough concentrations are expressed as geometric means with 90% CIs, and represented as proportion of participants above the PA-IC_90_ (0 × 064 μg/mL).^21^ Participants with dolutegravir trough concentrations below the lower limit of quantitation (0 × 03 μg/mL) were included in the primary analysis and imputed as 50% lower limit of quantitation (0 × 015 μg/mL). We did a sensitivity analysis excluding samples below the lower limit of quantitation.

For the safety analysis, we report the proportions of participants with grade 3 or 4 adverse events in either arm and all serious adverse events. ISI results were expressed as proportion of participants with any treatment-emergent insomnia (at least subthreshold insomnia, ISI questionnaire score ≥8) after baseline and MMS results as the proportion of participants with a change in score of at least one point from baseline.

Statistical analyses were done with Stata (version 16.1). The study is registered with ClinicalTrials.gov, NCT03851588.

### Role of the funding source

The funder of the study had no role in study design, data collection, data analysis, data interpretation, or writing of the report.

## Results

**Figure 1:**
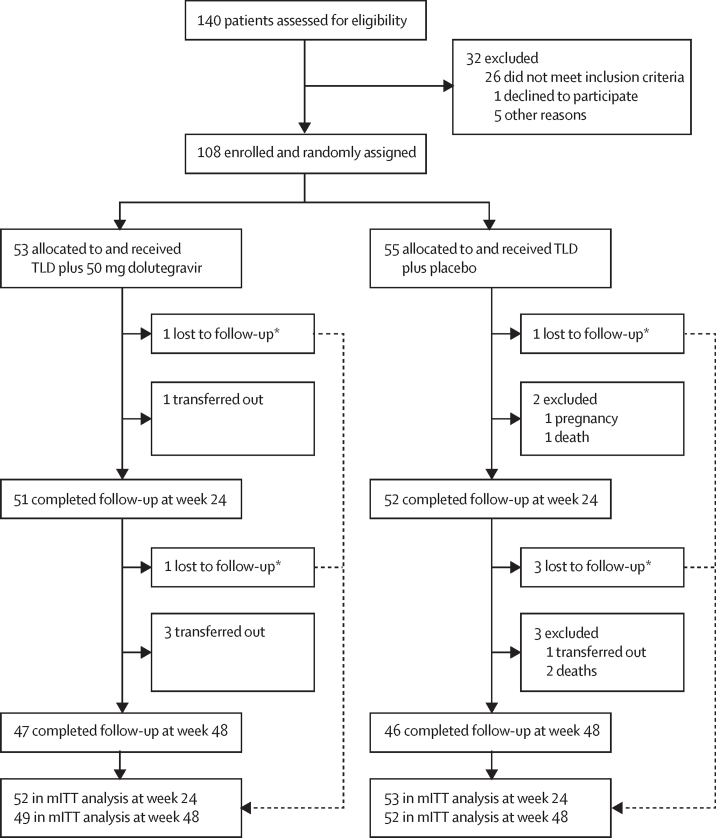
Trial profile TLD=tenofovir disoproxil fumarate, lamivudine, and dolutegravir. mITT=modified intention to treat. *As per the US Food and Drug Administration snapshot approach, patients lost to follow-up were included in the mITT analysis and were considered to have virological failure.

Between Nov 28, 2019, and July 23, 2021, we screened 140 patients for the study (figure 1). 108 patients were enrolled and randomly assigned to tenofovir disoproxil fumarate, lamivudine, and dolutegravir plus supplemental 50 mg dolutegravir (n=53) or tenofovir disoproxil fumarate, lamivudine, and dolutegravir plus matched placebo (n=55). All randomly assigned participants received at least one dose of the study medication and were included in the modified intention-to-treat analysis (appendix pp 1–2).

Baseline characteristics were similarly distributed between arms (table 1). 35% (38/108) of participants identified as female and 65% (70/108) as male. Most participants were ART-naive at enrolment. Median time from starting antituberculosis therapy until ART initiation (enrolment) was 8 weeks (IQR 6·6–8·6). Median time on antituberculosis therapy after ART initiation was 16 weeks (14·1–19·4). Median increase in weight at weeks 24 and 48 was similar by arm (appendix p 3).

**Table 1:** Baseline characteristics of participants by study arm

			**Supplemental dolutegravir arm (n=53)**	**Placebo arm (n=55)**	**Total (n=108)**
**Demographics**					
Age, years			33 (28–38)	37 (33–44)	35 (31–40)
Sex					
	Female		19 (36%)	19 (35%)	38 (35%)
	Male		34 (64%)	36 (65%)	70 (65%)
Weight, kg			56 (51–62)	55 (51–62)	56 (51–62)
BMI, kg/m^2^			20·0 (18·7–22·3)	20·2 (18·3–22·8)	20·1 (18·5–22·6)
**HIV characteristics**					
HIV-1 RNA log_10_(log_10_ copies per mL)			5·1 (4·6–5·6)	5·2 (4·6–5·7)	5·2 (4·6–5·7)
	≤100 000 copies per mL, n (%)		21 (40%)	21 (38%)	42 (39%)
	>100 000 copies per mL, n (%)		32 (60%)	34 (62%)	66 (61%)
CD4 count (cells per μL)			197 (145–260)	183 (145–316)	188 (145–316)
	≤200		27 (51%)	30 (55%)	57 (53%)
	>200		26 (49%)	25 (45%)	51 (47%)
Baseline ART status, n (%)					
	ART naive		44 (83%)	44 (80%)	88 (81%)
	First-line ART interrupted		9 (17%)	11 (20%)	20 (19%)
		On ART <6 months before interruption	8 (15%)	5 (9%)	13 (12%)
		On ART ≥6 months before interruption	1 (2%)	6 (11%)	7 (6%)
**Tuberculosis characteristics**					
Diagnosis					
	Microbiological or histological (or both)		38 (72%)	39 (71%)	77 (71%)
	Clinical or radiological (or both)		15 (28%)	16 (29%)	31 (29%)
Site					
	Pulmonary		40 (75%)	37 (67%)	77 (71%)
	Extrapulmonary		10 (19%)	13 (24%)	23 (21%)
	Disseminated		3 (6%)	5 (9%)	8 (7%)
Weeks on tuberculosis treatment at enrolment			8·0 (7·0–8·9)	8·0 (6·0–8·3)	8·0 (6·6–8·6)

Data are median (IQR) or n (%). Baseline was date of screening. ART=antiretroviral therapy.

**Figure 2:**
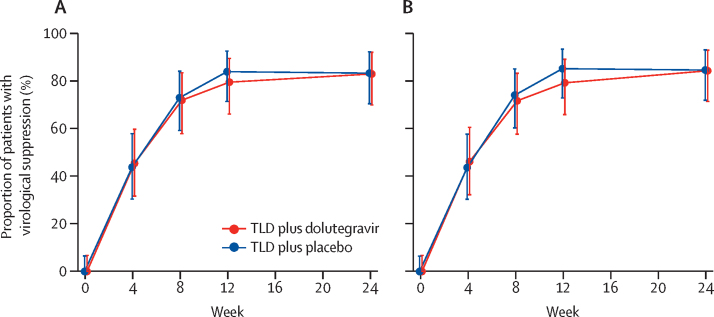
Virological suppression (HIV-1 RNA <50 copies per mL) over time Modified intention-to-treat (A) and per-protocol (B) analysis. The points and error bars show the proportion (95% CI). TLD=tenofovir disoproxil fumarate, lamivudine, and dolutegravir.

The primary outcome, virological suppression at week 24 by modified intention to treat, was acceptable, with the lower bound of the 95% CI equal to our predefined margin of 70% in both arms (table 2; figure 2). Six of nine participants who did not have virological suppression at week 24 in the supplemental dolutegravir arm and five of nine participants in the placebo arm had low level viraemia (50–999 copies per mL appendix p 1). Among ART-naive participants, 36 (84%, 95% CI 69–93) of 43 in the supplemental dolutegravir arm and 36 (86%, 71–95) of 42 in the placebo arm had virological suppression; compared with seven (78%, 40–97) of nine patients in the supplementary dolutegravir arm and eight (73%, 39–94) of 11 in the placebo arm (appendix p 2).

**Table 2:** Proportion of participants with virological suppression (HIV-1 RNA <50 copies per mL)

		**Supplemental dolutegravir arm (n=53)**	**Placebo arm (n=55)**
Week 24*			
	Modified intention to treat	43/52 (83%, 70–92)	44/53 (83%, 70–92)
	Per protocol	43/51 (84%, 71–93)	44/52 (85%, 72–93)
Week 12†			
	Modified intention to treat	42/53 (79%, 66–89)	46/55 (84%, 71–92)
	Per protocol	42/53 (79%, 66–89)	46/54 (85%, 73–93)
Week 48†			
	Modified intention to treat	34/49 (69%, 55–82)	35/52 (67%, 53–80)
	Per protocol	34/47 (72%, 57–84)	35/46 (76%, 61–87)

Data are n/N (%, 95% CI).

* Primary endpoint.

† Secondary endpoint.

The proportion of participants with virological suppression at week 48 decreased in both arms from week 24 (table 2). Secondary outcomes at week 48 by arm are shown in the appendix (p 4).

Participants with study-defined virological failure were similarly distributed between groups (appendix p 5). All participants with virological failure had genotypic antiretroviral resistance testing, except one participant for whom the testing analysis was not successful and subsequent HIV-1 RNA measurements were suppressed. No participant with virological failure had treatment-emergent integrase strand transfer inhibitor resistance (appendix pp 5–6). One participant had potential low-level resistance to integrase strand transfer inhibitor (raltegravir and elvitegravir; appendix pp 5–6), but these mutations were present at baseline.

Adherence as assessed by tenofovir diphosphate concentrations in dried blood spots was similar between the study arms at weeks 12, 24, and 48, but decreased from week 24 to week 48 (table 3; appendix p 13). Tenofovir diphosphate concentrations were independently associated with virological suppression when adjusted for sex at week 24 (p=0·033) and at week 48 (p=0·0079; appendix p 7). When patients from the study arms were aggregated, poor adherence (tenofovir diphosphate concentration <350 fmol/punch) was detected in four (4%) of 103 patients at week 24 compared with 12 (13%) of 93 patients at week 48 (p=0·011). Tenofovir diphosphate dried blood spot results stratified by virological suppression at weeks 24 and 48 showed similar adherence between arms among participants who did not have virological suppression (appendix p 8).

**Table 3:** Participant adherence at weeks 12, 24, and 48

		**Supplemental dolutegravir arm (n=53)**		**Placebo arm (n=55)**	
		Female (n=19)	Male (n=34)	Female (n=19)	Male (n=36)
**Week 12**					
n		19	33	19	34
Tenofovir diphosphate, fmol/punch		1222 (810–1846)	1333 (756–1863)	1268 (708–2165)	1239 (871–1959)
Adherence category, fmol/punch					
	<350	1 (5%)	2 (6%)	0	1 (3%)
	350–700	1 (5%)	6 (18%)	4 (21%)	5 (15%)
	701–1250	8 (42%)	8 (24%)	5 (26%)	11 (32%)
	>1250	9 (47%)	17 (52%)	10 (53%)	17 (50%)
**Week 24**					
n		18	33	17	35
Tenofovir diphosphate (fmol/punch)		1281 (1113–1623)	1491 (858–2124)	1449 (1003–1708)	1479 (857–1997)
Adherence category, fmol/punch					
	<350	0	2 (6%)	1 (6%)	1 (3%)
	350–700	0	2 (6%)	2 (12%)	5 (14%)
	701–1250	9 (50%)	11 (33%)	3 (18%)	7 (20%)
	>1250	9 (50%)	18 (55%)	11 (65%)	22 (63%)
**Week 48**					
n		16	31	16	30
Tenofovir diphosphate (fmol/punch)		1424 (1039–2514)	1380 (635–2625)	1502 (920–2658)	1573 (1105–1981)
Adherence category, fmol/punch					
	<350	1 (6%)	5 (16%)	3 (19%)	3 (10%)
	350–700	1 (6%)	4 (13%)	0	2 (7%)
	701–1250	5 (31%)	4 (13%)	4 (25%)	5 (17%)
	>1250	9 (56%)	18 (58%)	9 (56%)	20 (67%)

Data are median (IQR). Adherence measured as tenofovir diphosphate concentrations in dried blood spots and presented as categories of presumed doses of medication taken per week. Adherence categories, <350 fmol/punch (equivalent in men: <1·2 doses per week and women: <0·6 doses per week), 350–700 fmol/punch (men: 1·2–3·2 doses per week and women: 0·6–2·0 doses per week), 701–1250 fmol/punch (men: 3·2–6·0 doses per week and women: 2·0–5·3 doses per week), and >1250 fmol/punch (men: >6·0 doses per week and women: >5·3 doses per week).

18 participants still on antituberculosis therapy (12 in the supplemental dolutegravir arm and six in the placebo arm) at week 24 were excluded from analysis of dolutegravir trough concentrations at weeks 4, 24, and 48 (appendix p 14). The primary analysis that imputed dolutegravir trough concentrations below the lower limit of quantification had more participants in the supplemental dolutegravir arm than the placebo arm, with dolutegravir trough concentrations above the PA-IC_90_ at week 4 (35 [90%] of 39 patients *vs* 28 [65%] of 43 patients; p=0·0083) and week 24 (32 [97%] of 33 patients *vs* 34 [81%] of 42 patients; p=0·034; appendix p 9). In the sensitivity analysis (excluding participants with dolutegravir trough concentrations below the lower limit of quantitation) more participants in the supplemental dolutegravir arm than in the placebo arm had dolutegravir trough concentrations above the PA-IC_90_ at week 4 (35 [100%] of 35 patients *vs* 28 [75%] of 36 patients; p=0·0051), but at weeks 24 and 48 all participants had dolutegravir trough concentrations above the dolutegravir PA-IC_90_ (appendix p 10).

Median CD4 count increase from baseline was 111 cells per μL in the supplemental dolutegravir arm and 101 cells per μL in the placebo arm at week 24, and 149 cells per μL in the supplemental dolutegravir arm and 163 cells per μL in the placebo arm at week 48.

Self-reported grade 3 or 4 insomnia events occurred more frequently in the supplemental dolutegravir arm than in the placebo arm (three [6%] of 53 patients *vs* no patients; p=0·12; table 4). One participant in the supplemental dolutegravir arm was classified as having developed mild immune reconstitution inflammatory syndrome, but did not meet our case definition. Five participants developed serious adverse events, all of whom were in the placebo arm, and none of these were deemed to be related to study medication (table 4). Although not statistically significant, treatment-emergent insomnia (at least subthreshold) determined by the ISI questionnaire was more common in the supplemental dolutegravir arm than in the placebo arm: ten (20%) of 51 patients versus four (8%) of 52 patients (p=0·069), but reduced over time on treatment (appendix pp 11, 15). Changes in MMS questionnaire score from baseline were similar by study arm to week 48 (appendix p 12).

**Table 4:** Clinical and laboratory grade 3 or 4 adverse events, serious adverse events, and deaths that occurred until week 48

		**Supplemental dolutegravir arm (n=53)**	**Placebo arm (n=55)**
Participants with clinical grade 3–4 adverse events		11 (21%)	10 (18%)
	Total number of clinical grade 3–4 adverse events*	13	18
Clinical grade 3–4 adverse events			
	Weight loss	2 (4%)	2 (4%)
	Insomnia	3 (6%)	0
	Pneumonia	2 (4%)	1 (2%)
	Nausea or vomiting (or both)	0	2 (4%)
	Gastritis	0	2 (4%)
	Rash	0	2 (4%)
	Trauma-related injury	2 (4%)	0
	Reactivation of or unsuccessfully treated tuberculosis	0	2 (4%)
	Acute renal impairment	0	2 (4%)
	Balanitis with paraphimosis	1 (2%)	0
	Bowel obstruction	0	1 (2%)
	Oesophageal candidiasis	0	1 (2%)
	Hyperkalaemia	1 (2%)	0
	Immune reconstitution inflammatory syndrome	1 (2%)	0
	Peripheral neuropathy	1 (2%)	0
	Prostatitis	0	1 (2%)
Drug-related clinical grade 3–4 adverse events†		5 (9%)	3 (5%)
Participants with serious adverse events		0	5 (9%)‡
Drug-related serious adverse events		0	1 (2%)
Deaths		0	3 (5%)
Participants with laboratory grade 3–4 adverse events		12 (23%)	13 (24%)
	Low CD4 count	9 (17%)	7 (13%)
	Low estimated glomerular filtration rate	2 (4%)	6 (11%)
	High potassium	1 (2%)	2 (4%)
	High alanine aminotransferase	0	1 (2%)

Data are n (%) or n. Data capture adverse events that took place at any point from the first dose of study drug until the end of week 48.

* Total number of adverse events.

† Drug-related clinical adverse events defined as at least possibly related to treatment.

‡ One participant was admitted to hospital with treatment-resistant oesophageal candidiasis, atrophic gastritis, and acute renal impairment and died secondary to presumed cardiac arrest; one participant died secondary to a trauma-related event; one participant was admitted to hospital with severe community-acquired pneumonia and had a subsequent readmission for nosocomial pneumonia, one participant was admitted to hospital with a lichenoid rash possibly related to antituberculosis therapy, and one participant was admitted to hospital with bowel obstruction and died shortly after corrective surgery.

## Discussion

In participants receiving treatment for both tuberculosis and HIV, we found acceptable virological suppression in the placebo arm and the supplemental dolutegravir arm at week 24. No participants developed treatment-emergent resistance mutations to integrase strand transfer inhibitors by week 48. Dolutegravir was generally well tolerated in both arms. Our findings suggest that supplemental dolutegravir dosing might not be necessary in patients on rifampicin-based antituberculosis therapy.

Virological suppression decreased from week 24 to week 48, which was probably a result of reduced adherence (supported by a higher proportion of participants with low tenofovir diphosphate). At week 24 participants were issued a 6-month supply of ART without follow-up visits between weeks 24 and 48 as per local guidelines, to reduce clinic burden during the COVID-19 epidemic, which could have accounted for reduced adherence.

Our finding that standard-dose dolutegravir is as effective as double-dose dolutegravir in people living with HIV on rifampicin-based antituberculosis therapy is consistent with findings of two other studies. A small trial in Thailand, which randomly assigned people with HIV with tuberculosis to dolutegravir once daily or twice daily, reported virological suppression in 18 of 20 patients in both groups.^22^ A retrospective cohort study in Botswana reported virological suppression during tuberculosis therapy in 204 (95%) of 214 patients on standard dolutegravir dosing and 241 (95%) of 254 patients who received supplemental dolutegravir dosing.^7^

A key question is whether a phase 3 trial is necessary to confirm our findings. The probable delay in policy change that running a phase 3 trial would entail is illustrated by the experience of researchers exploring different dosing strategies for the first-generation integrase strand transfer inhibitor raltegravir in people with HIV on rifampicin-based antituberculosis therapy. Plasma concentrations of raltegravir are substantially reduced by rifampicin and double dosing is recommended.^23^ In a phase 2 non-comparative randomised trial (ANRS 12 180 Reflate TB) at week 24, virological suppression was achieved in 39 patients (76% [95% CI 65–88]) of 51 in the raltegravir 400 mg group, 40 patients (78% [67–90]) in the raltegravir 800 mg group, and 32 patients (63% [49–76]) in the efavirenz group.^24^ The same investigator group did a phase 3 non-inferiority, randomised trial (ANRS 12300 Reflate TB 2) comparing raltegravir standard dose with efavirenz in participants with tuberculosis.^25^ Virological suppression was similar in both arms at week 24 but at week 48, the primary endpoint was reached in 140 (61%) of 230 participants in the raltegravir group and 150 (66%) of 227 patients in the efavirenz arm; raltegravir did not meet the non-inferiority criterion.^25^ The phase 3 raltegravir study was published 7 years after the phase 2 study.

None of our participants with study-defined virological failure developed treatment-emergent resistance mutations to dolutegravir. Our findings are in keeping with the INSPIRING study, in which no acquired resistance mutations occurred in the dolutegravir arm.^6^ By contrast, 12 (38%) of 32 participants with protocol-defined virological failure in ANRS 12300 Reflate TB 2 developed resistance to raltegravir.^25^ These findings reflect that dolutegravir has a higher genetic barrier to resistance than does raltegravir.

In our study, solicited and self-reported insomnia events occurred earlier and more frequently in the supplemental dolutegravir arm than in the placebo arm, but these differences were not statistically significant. Dolutegravir treatment is associated with an increased risk of insomnia^26^ and the higher early incidence of insomnia we observed in the supplemental dolutegravir arm could result from higher dolutegravir exposure. However, findings of an association between increasing dolutegravir exposure and worsening sleep quality have been contradictory.^27^, ^28^, ^29^

Our study has limitations. First, the study was not powered to make efficacy comparisons between arms. Second, our findings might not be generalisable to people on dolutegravir-based second-line ART or to people with CD4 counts below 100 cells per μL. Third, we only enrolled participants who were ART-naive or had interrupted first-line ART without virological failure. Our findings might not be generalisable to people with dolutegravir in second-line ART. Fourth, we did not detect treatment-emergent dolutegravir resistance in the placebo arm, but larger sample sizes will be needed to determine the risk of resistance. Fifth, more participants in the placebo arm had dolutegravir trough concentrations below the lower limit of quantitation at weeks 4 and 24. However, as we did not observe the previous dose, we cannot be sure that the plasma sample was taken in the specified time window. Sixth, our study was done at a clinical research trial site; virological outcomes might be worse in real-world settings.

In conclusion, we found similar rates of virological suppression in the supplemental dolutegravir and placebo arms when prescribed with rifampicin-based antituberculosis therapy, and there were no treatment-emergent integrase strand transfer inhibitor resistance mutations among participants with study-defined virological failure. Our findings suggest that twice-daily dolutegravir dosing might be unnecessary in people with HIV-associated tuberculosis. More evidence, from cohort studies or possibly a phase 3 trial, might be necessary to change policy on the need for a supplemental dolutegravir dose with rifampicin-based antituberculosis therapy.

## Data sharing

Study data will not be publicly available. Data can be made available by the corresponding author to any researcher interested. Deidentified participant data and a data dictionary can be made available and shared under a data transfer agreement. Requests for access to the RADIANT-TB study data should be sent to gary.maartens@uct.ac.za. The study protocol, statistical analysis plan, and informed consent have been published online.

## Declaration of interests

BS did statistical analysis for HIV-related and tuberculosis-related clinical trials for MetaVirology. GMe lectured for Gilead Sciences and participated in a data safety monitoring board or advisory board for Otsuka. All other authors declare no competing interests.
