# Supplementary material for: Standard-dose versus double-dose dolutegravir in HIV-associated tuberculosis in South Africa (RADIANT-TB): a phase 2, non-comparative, randomised controlled trial
Source: Lancet HIV. 2023 May 22;10(7):e433–41. doi: 10.1016/S2352-3018(23)00081-4 (PMC10322729; doi:10.1016/S2352-3018(23)00081-4)

THE LANCET
HIV

Supplementary appendix

This appendix formed part of the original submission and has been peer reviewed. We post it as supplied by the authors.

Supplement to: Griesel R, Zhao Y, Simmons B, et al. Standard-dose versus double-dose dolutegravir in HIV-associated tuberculosis in South Africa (RADIANT-TB): a phase 2, non-comparative, randomised controlled trial. *Lancet HIV* 2023; published online May 22. https://doi.org/10.1016/S2352-3018(23)00081-4.

Supplemental Table 1: Primary outcome analysis at week 24 participant classification by modified intention to treat using US Food and Drug Administration snapshot approach.

	**Supplemental DTG arm (n=53)**	**Placebo arm (n=55)**
Included in mITT population at Week 24	*52 (98)*	*53 (96)*
**HIV-1 RNA <50 copies/mL**	**43 (83)**	**44 (83)**
**HIV-1 RNA ≥50 copies/mL***	**9 (17)**	**9 (17)**
50-399	6 (12)	4 (8)
400-999	0	1 (2)
≥1,000	2 (4)	3 (6)
d/c with lack/loss of efficacy[a]	1 (2)	1 (2)
**No virologic data at Week 24 window**	**0**	**0**
d/c due to AE or death[†]	0	0
d/c for other reasons[#]	0	0
On study but missing data in window	0	0
Excluded from mITT population at Week 24	*1 (2)*	*2 (4)*
Pregnant	0	1
Transferred out	1	0
Death (non-HIV/non-drug cause)	0	1

mITT population includes all participants receiving at least one dose, excluding those: switching study drug for reasons of stopping contraception or wish to become pregnant, becoming pregnant, transferred out for non-clinical reasons, and deaths from non-HIV and non-drug causes.

*Includes participants who: had an HIV-1 RNA ≥50 copies/mL in the 24-week window; who discontinued study drugs or the study before Week 24 for lack or loss of efficacy (virology first hierarchy); or who switched any background therapy not permitted per protocol

†Includes participants who discontinued because of AE or death at any time point from Day 1 through the time window if this resulted in no virologic data during the week 24 window (regardless of HIV-1 RNA result at time of d/c)

aParticipants discontinuing study/study drug before week 24 for reasons other than AE or death and whose last HIV-1 RNA was ≥50 copies/mL

#Other reasons include: withdrew consent, loss to follow-up, among others (only participants who achieved virologic suppression can be counted as d/c for other reasons)

d/c = discontinued, AE = adverse event, mITT = modified intention to treat

Supplemental Table 2: Proportion of participants with HIV-1 RNA <50 copies/mL by baseline antiretroviral therapy (ART) naïve or first-line ART interruption status at primary and secondary time points by modified intention to treat and per protocol analysis

	**Supplemental DTG arm (n=53)**	**Placebo arm (n=55)**
**ART naïve (n=88)**	44	45
Week 48		
mITT*	29/40<br>73% (56-85%)	27/41<br>66% (49-80%)
PP†	29/39<br>74% (58-87%)	27/36<br>75% (58-88%)
Week 24		
mITT*	36/43<br>84% (69-93%)	36/42<br>86% (71-95%)
PP†	36/42<br>86% (71-95%)	36/42<br>86% (71-95%)
Week 12		
mITT*	36/44<br>82% (67-92%)	36/44<br>82% (67-92%)
PP†	36/44<br>82% (67-92%)	36/43<br>84% (69-93%)
**First-line ART interrupted (n=20)**	9	11
Week 48		
mITT*	5/9<br>56% (21-86%)	8/11<br>73% (39-94%)
PP†	5/8<br>63% (24-91%)	8/10<br>80% (44-97%)
Week 24		
mITT*	7/9<br>78% (40-97%)	8/11<br>73% (39-94%)
PP†	7/9<br>78% (40-97%)	8/10<br>80% (44-97%)
Week 12		
mITT*	6/9<br>67% (30-93%)	10/11<br>91% (59-100%)
PP†	6/9<br>67% (30-93%)	10/11<br>91% (59-100%)

*mITT population includes all participants receiving at least one dose, excluding individuals: switching study drug for reasons of stopping contraception or wish to become pregnant, becoming pregnant, transferred out for non-clinical reasons, and deaths from non-HIV and non-drug causes.

†PP population is as the mITT population, additionally excluding those lost to follow-up, those with missing HIV-1 RNA within the window, and participants who stopped or were change from the study drug for reasons other than failure of the regimen.

ART = antiretroviral therapy, mITT = modified intention to treat, PP = per protocol

Supplemental Table 3: Change in weight from baseline to weeks 24 and 48 stratified by arm and total cohort

	**Supplemental DTG arm (n=53)**		**Placebo arm (n=55)**		**Total (n=108)**	
	**n**	**Median (IQR)**	**n**	**Median (IQR)**	**n**	**Median (IQR)**
**Baseline weight**	53	55·9 (51·0-61·9)	55	55·2 (51·0-62·3)	108	55·7 (51·0-62·3)
**Week 24**						
Weight	51	60·3 (56·6-67·7)	52	60·7 (56·0-68·2)	103	60·4 (56·4-67·8)
Change from baseline	51	5·4 (2·5-7·3)	52	4·8 (2·2-9·4)	103	4·9 (2·4-9·0)
**Week 48**						
Weight	47	61·9 (57·3-69·0)	46	63·3 (55·6-71·3)	93	62·1 (56·3-69·0)
Change from baseline	47	5·7 (1·6-11·4)	46	7·8 (2·5-12·6)	93	6·3 (1·9-11·7)

Supplemental Table 4: Secondary outcome analysis at week 48 by modified intention to treat using US Food and Drug Administration snapshot approach.

	**Supplemental DTG arm (n=53)**	**Placebo arm (n=55)**
Included in mITT population at Week 48	*49 (92)*	*52 (95)*
**HIV-1 RNA <50 copies/mL**	**34 (69)**	**35 (67)**
**HIV-1 RNA ≥50 copies/mL***	**14 (29)**	**14 (27)**
50-399	6 (12)	3 (6)
400-999	1 (2)	2 (4)
≥1,000	6 (12)	6 (12)
d/c with lack/loss of efficacy[a]	1 (2)	3 (6)
**No virologic data at Week 48 window**	**1 (2)**	**3 (6)**
d/c due to AE or death[†]	0	2 (4)
d/c for other reasons[#]	1 (2)	1 (2)
On study but missing data in window	0	0
Excluded from mITT population at Week 48	*4 (8)*	*3 (5)*
Pregnant	0	1
Transferred out	4	1
Death (non-HIV/non-drug cause)	0	1

mITT population includes all participants receiving at least one dose, excluding those: switching study drug for reasons of stopping contraception or wish to become pregnant, becoming pregnant, transferred out for non-clinical reasons, and deaths from non-HIV and non-drug causes.

*Includes participants who: had an HIV-1 RNA ≥50 copies/mL in the 48-week window; who discontinued study drugs or the study before Week 48 for lack or loss of efficacy (virology first hierarchy); or who switched any background therapy not permitted per protocol

†Includes participants who discontinued because of AE or death at any time point from Day 1 through the time window if this resulted in no virologic data during the week 48 window (regardless of HIV-1 RNA result at time of d/c)

aParticipants discontinuing study/study drug before week 48 for reasons other than AE or death and whose last HIV-1 RNA was ≥50 copies/mL

#Other reasons include: withdrew consent, loss to follow-up, among others (only participants who achieved virologic suppression can be counted as d/c for other reasons)

d/c = discontinued, AE = adverse event, mITT = modified intention to treat

Supplemental Table 5: Listing of individual participant data and outcomes among participants with study defined virological failure and genotypic antiretroviral resistance testing showing resistance mutations

PID	Week of VF	Resistance test result at baseline	Resistance test result at VF	Management and outcome
**Supplemental dolutegravir arm (n=53)**				
0036	W24	N/A	High-level resistance to efavirenz, nevirapine, and rilpivirine. Intermediate-level resistance to etravirine.	Enhanced adherence counselling and continue TLD. Repeat VL LDL at W28.
0065	W48	N/A	Potential low-level resistance to atazanavir, lopinavir, saquinavir, fosamprenavir, and indinavir.	Enhanced adherence counselling and continue TLD. No repeat VL after W48.
0086	W28	N/A	Potential low-level resistance to efavirenz, etravirine, nevirapine, and rilpivirine. High-level resistance to emtricitabine and lamivudine.	Enhanced adherence counselling and continue TLD. Repeat VL LDL at W36.
**Placebo arm (n=55)**				
0052	W48	N/A	High-level resistance to efavirenz and nevirapine only.	Enhanced adherence counselling and continue TLD. No repeat VL after W48.
0076	W8	N/A	High-level resistance to efavirenz, etravirine, nevirapine, and rilpivirine only.	Enhanced adherence counselling and continue TLD. Repeat VL LDL at W20.
0133	W24	Potential low-level resistance to elvitegravir and raltegravir.	Potential low-level resistance to elvitegravir and raltegravir.	Previously suppressed at W20. Lost to follow-up after W24

Virologic failure is defined as HIV-1 RNA >1,000 copies/mL at Week 24 or participants who suppress their HIV-1 RNA <50 copies/mL and then subsequently rebound to an HIV-1 RNA >1,000 copies/mL
PID = persistent identifier, VF = virological failure, N/A = not applicable, TLD = tenofovir/emtricitabine/dolutegravir, VL = viral load (HIV-1 RNA), LDL = lower than detectable limit, W = week

Supplemental Table 6: Logistic regression assessing tenofovir-diphosphate dried blood spot concentrations for association with virologic suppression (HIV-1 RNA <50 copies/mL) at weeks 24 and 48

	**Week 24**				**Week 48**			
	**Univariable analysis**		**Multivariable analysis**		**Univariable analysis**		**Multivariable analysis**	
	**OR (95% CI)**	**P-value**	**OR (95% CI)**	**P-value**	**OR (95% CI)**	**P-value**	**OR (95% CI)**	**P-value**
**TFV-DP (fmol/punch) per 100-unit increase**	1·118 (1·010-1·237)	0·031	1·119 (1·009-1·242)	0·033	1·158 (1·040-1·290)	0·008	1·158 (1·039-1·292)	0·008
**Sex**								
**Female**	Referent group				Referent group			
**Male**	1·273 (0·444-3·645)	0·653	1·223 (0·390-3·831)	0·730	0·689 (0·275-1·725)	0·426	0·721 (0·237-2·191)	0·564

OR = odds ratio, TFV-DP = tenofovir diphosphate

Supplemental Table 7: Adherence as measured by tenofovir-diphosphate dried blood spot concentrations and stratified by virological suppression at weeks 24 and 48

	**Supplemental dolutegravir arm (n=53)**		**Placebo arm (n=55)**	
	Virologically suppressed (HIV-1 RNA <50 copies/mL)	Virologically unsuppressed (HIV-1 RNA ≥50 copies/mL)	Virologically suppressed (HIV-1 RNA <50 copies/mL)	Virologically unsuppressed (HIV-1 RNA ≥50 copies/mL)
**Week 24**				
n	43	8	44	8
TFV-DP (fmol/punch), median (IQR)	1446·7 (968·7-1890·7)	1147·2 (755·0-1902·1)	1547·8 (1135·2-2036·9)	847·1 (505·1-1068·6)
Adherence category, n(%)				
<350	1 (2)	1 (13)	0 (0)	2 (25)
350-700	2 (5)	0 (0)	6 (14)	1 (13)
701-1250	16 (37)	4 (50)	7 (16)	3 (38)
>1250	24 (56)	3 (38)	31 (70)	2 (25)
**Week 48**				
n	34	13	35	11
TFV-DP (fmol/punch), median (IQR)	1637·3 (1211·8-2856·6)	648·0 (157·4-1009·9)	1693·2 (1207·6-2875·1)	348·0 (48·8-1345·1)
Adherence category, n(%)				
<350	1 (3)	5 (39)	0 (0)	6 (55)
350-700	3 (9)	2 (15)	1 (3)	1 (9)
700-1250	6 (18)	3 (23)	8 (23)	1 (9)
>1250	24 (70)	3 (23)	26 (74)	3 (27)

Adherence categories: <350 fmol/punch (equivalent of men: <1·2 doses per week and women: <0·6 doses per week), 350-700 fmol/punch (equivalent of men: 1·2 - 3·2 doses per week and women: 0·6-2·0 doses per week), 701-1250 fmol/punch (equivalent of men: 3·2-6 doses per week and women: 2·0-5·3 doses per week) and >1250 fmol/punch (men: >6 doses per week and women: >5·3 doses per week).
TFV-DP = tenofovir diphosphate

Supplemental Table 8: Dolutegravir trough concentrations at week 4 (participants on supplemental dolutegravir or placebo included) and weeks 24 and 48 (participants on supplemental dolutegravir or placebo excluded: week 24 had 18 exclusions [12 in the supplemental dolutegravir arm and 6 in the supplemental placebo arm] and week 48 had 0 exclusions) by arm including all participants with dolutegravir trough concentrations below the limit of quantification

	**Supplemental dolutegravir arm (n=53)**			**Placebo arm (n=55)**		
	C12h Week 4	C24h Week 24	C24h Week 48	C12h Week 4	C24h Week 24	C24h Week 48
n	39	33	36	43	42	37
Geometric mean (90% CI)	0·498 (0·342-0·726)	0·911 (0·653-1·270)	0·414 (0·245-0·702)	0·103 (0·074-0·144)	0·409 (0·254-0·656)	0·471 (0·278-0·798)
BLLQ, n(%)	4 (10)	1 (3)	7 (19)	7 (16)	8 (21)	7 (19)
Above PA-IC_{90}, n(%)	35 (90)	32 (97)	29 (81)	28 (65)	34 (81)	30 (81)

Data are for the dolutegravir treatment troughs (C12h ±2 hours of supplementary dolutegravir/placebo dose at Week 4 and C24h ±4 hours of last tenofovir/lamivudine/dolutegravir dose at Week 24 and Week 48; µg/mL).
Individuals still taking supplemental dolutegravir or placebo at weeks 24 (18 participants [12 in the supplemental dolutegravir arm and 6 in the supplemental placebo arm]) and 48 (0 participants) were excluded from the analyses.

The LLQ was 0.03 µg/mL; observations at the LLQ were imputed as 0·015 µg/mL (i.e., the midpoint between 0 and the LLQ).

The dolutegravir PA-IC_{90} was equal to 0·064 µg/mL.
LLQ = lower limit of quantification, PA-IC_{90} = protein adjusted 90% inhibitory concentration for dolutegravir, BLLQ = below the lower limit of quantification

Supplemental Table 9: Dolutegravir trough concentrations at week 4 (participants on supplemental dolutegravir or placebo included) and weeks 24 and 48 (participants on supplemental dolutegravir or placebo excluded: week 24 had 18 exclusions [12 in the supplemental dolutegravir arm and 6 in the supplemental placebo arm] and week 48 had 0 exclusions) by arm and excluding all participants with dolutegravir trough concentrations below the limit of quantification

	**Supplemental dolutegravir arm (n=53)**			**Placebo arm (n=55)**		
	C12h Week 4	C24h Week 24	C24h Week 48	C12h Week 4	C24h Week 24	C24h Week 48
n	35	32	29	36	34	30
Geometric mean (90% CI)	0·743 (0·599-0·923)	1·035 (0·799-1·342)	0·923 (0·679-1·256)	0·150 (0·112-0·202)	0·889 (0·679-1·166)	1·053 (0·778-1·425)
Above PA-IC_{90}, n(%)	35 (100)	32 (100)	29 (100)	28 (78)	34 (100)	30 (100)

Data are for the dolutegravir treatment troughs (C12h ±2 hours of supplementary dolutegravir/placebo dose at Week 4 and C24h ±4 hours of last tenofovir/lamivudine/dolutegravir dose at Week 24 and Week 48; µg/mL).
Individuals still taking supplemental dolutegravir or placebo at weeks 24 (18 participants [12 in the supplemental dolutegravir arm and 6 in the supplemental placebo arm]) and 48 (0 participants) were excluded from the analyses.
Participants with values below the LLQ were excluded from analyses at weeks 4 (11 participants [4 in the supplemental dolutegravir arm and 7 in the supplemental placebo arm], 24 (9 participants [1 in the supplemental dolutegravir arm and 8 in the supplemental placebo arm]) and 48 (14 participants [7 in the supplemental dolutegravir arm and 7 in the supplemental placebo arm]) due to likely poor adherence.

The dolutegravir PA-IC_{90} was equal to 0·064 µg/mL.
LLQ = lower limit of quantification, PA-IC_{90} = protein adjusted 90% inhibitory concentration for dolutegravir

Supplemental Table 10: Insomnia severity index questionnaire results at each assessed time point

Assessment	Supplemental dolutegravir arm (n=53)	Placebo arm (n=55)
**ISI, n(%)**		
**Baseline**	*53*	*55*
No insomnia	51 (96)	52 (95)
Subthreshold insomnia	1 (2)	2 (4)
Moderate insomnia	1 (2)	1 (2)
**Week 4**	*53*	*55*
No insomnia	47 (89)	52 (95)
Subthreshold insomnia	3 (6)	3 (5)
Moderate insomnia	3 (6)	-
**Week 8**	*53*	*53*
No insomnia	51 (96)	51 (96)
Subthreshold insomnia	2 (4)	2 (4)
Moderate insomnia	-	-
**Week 12**	*53*	*54*
No insomnia	53 (100)	53 (98)
Subthreshold insomnia	-	1 (2)
Moderate insomnia	-	-
**Week 16**	*52*	*53*
No insomnia	50 (96)	52 (98)
Subthreshold insomnia	2 (4)	1 (2)
Moderate insomnia	-	-
**Week 20**	*52*	*53*
No insomnia	51 (98)	53 (100)
Subthreshold insomnia	1 (2)	-
Moderate insomnia	-	-
**Week 24**	*51*	*52*
No insomnia	50 (98)	52 (100)
Subthreshold insomnia	1 (2)	-
Moderate insomnia	-	-
**Week 48**	*47*	*46*
No insomnia	47 (100)	45 (98)
Subthreshold insomnia	-	1 (2)
Moderate insomnia	-	-
**Any insomnia after baseline***	11/53 (21)	6/55 (11)
**Any treatment emergent insomnia after baseline****	10/51 (20)	4/52 (8)

*Any ISI >7 until Week 48 (indicative of at least subthreshold insomnia)
**Any ISI >7 until Week 48 in participants with a baseline ISI ≤7
ISI scored from 0-28. Score categories: 0-7, no clinical significant insomnia; 8-14, subthreshold insomnia; 15-21, clinical insomnia (moderate severity); 22-28, clinical insomnia (severe).
ISI was measured at baseline & 4-weekly intervals until Week 24.
During data collection, one question was answered on a 0-5 scale. To keep within the 0-28 range, the scores for this question were rescaled as: 0=0; 1=1; 2=1; 3=2; 4=3; 5=4 (no participant scored 5).
ISI = insomnia severity index

Supplemental Table 11: Modified MINI screen questionnaire results at each time point and change from baseline

Assessment	Supplemental dolutegravir arm (n=53)	Placebo arm (n=55)
**MMS (20-item), median (IQR)**		
Baseline	0 (0-0)	0 (0-0)
Week 12	0 (0-0)	0 (0-0)
Week 24	0 (0-0)	0 (0-0)
Week 48	0 (0-0)	0 (0-0)
MMS increased from baseline[†], n(%)	2 (4)	4 (7)
MMS decreased from baseline[†], n(%)	5 (9)	6 (11)

[†]MMS score increased/decreased from baseline by at least one point at any point until Week 48
MMS includes 22 questions answered yes/no; 2 questions related to experiencing a traumatic event were excluded for a 20 point scale.
MMS was measured at baseline, week 12, and week 24
MMS = modified MINI screen

Supplemental Figure 1: Proportion of participants by insomnia severity index (ISI) category (A) at each time point from baseline to week 48 and (B) excluding participants with insomnia at baseline (ISI >7). A score 0-7 is classified as no clinically significant insomnia, 8-14 as subthreshold insomnia, 15-21 as moderate clinical insomnia, and 22-28 as severe clinical insomnia.

A

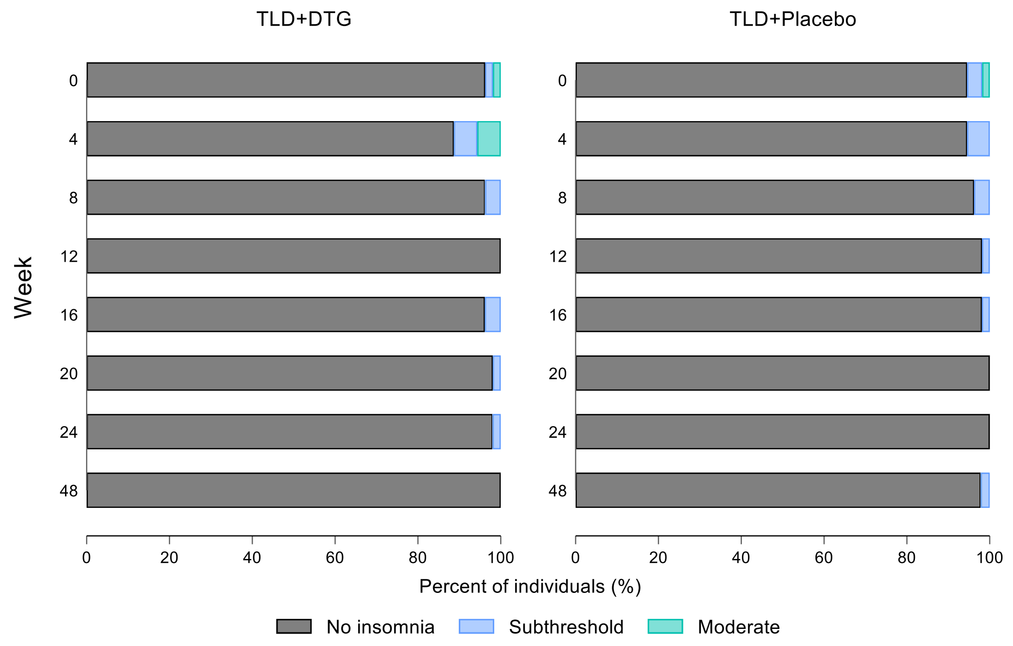

B

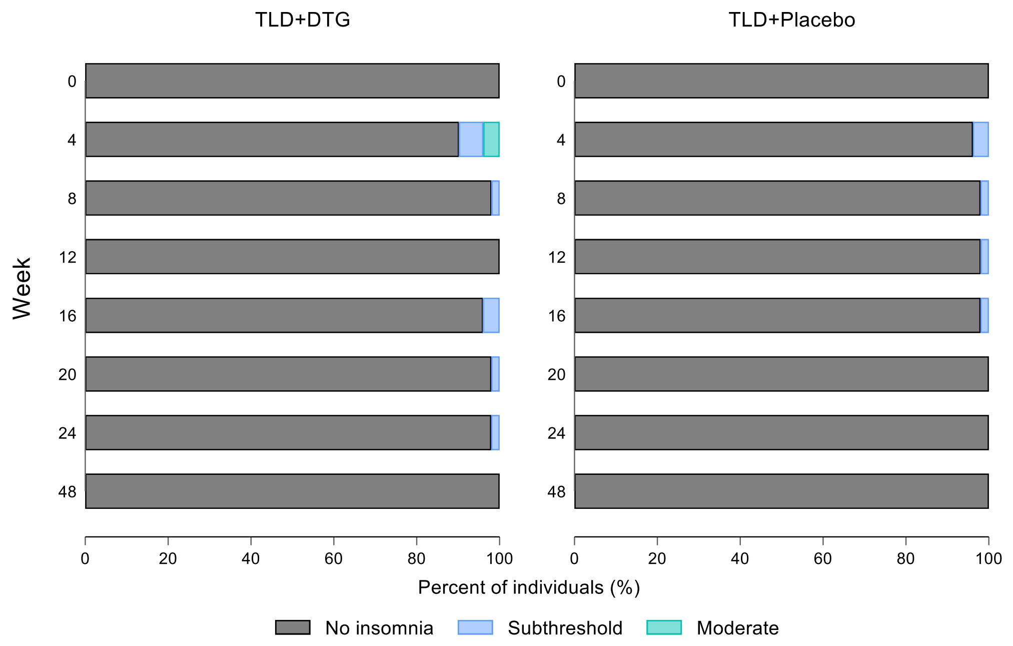

Supplement: Supplementary appendix [file mmc1.pdf]
